# The silent signals: emerging safety concerns in bispecific antibody therapy for multiple myeloma

**DOI:** 10.3389/fmed.2025.1593405

**Published:** 2025-08-13

**Authors:** Xiaoling Zhou, Erdan Luo, Wei Chen, Yi Deng, Bo Wu, Xia Jiang, Kaili Zhang, Fan Lai

**Affiliations:** ^1^Department of Pharmacy, Chengdu Women’s and Children’s Central Hospital, School of Medicine, University of Electronic Science and Technology of China, Chengdu, China; ^2^Department of Good Clinical Practice, Chengdu Women’s and Children’s Central Hospital, School of Medicine, University of Electronic Science and Technology of China, Chengdu, China; ^3^Department of Traditional Chinese Medicine, Chengdu Women’s and Children’s Central Hospital, School of Medicine, University of Electronic Science and Technology of China, Chengdu, China; ^4^Department of Obstetrics, Chengdu Women’s and Children’s Central Hospital, School of Medicine, University of Electronic Science and Technology of China, Chengdu, China; ^5^Graduate School of Human-Environment Studies, Kyushu University, Fukuoka, Japan

**Keywords:** bispecific antibodies, AEs, MM, pharmacovigilance, FAERS

## Abstract

**Background:**

Bispecific antibodies (BsAbs) are widely used for the treatment of multiple myeloma (MM), but their long-term safety still provokes concerns.

**Methods:**

Adverse event (AE) data on teclistamab, talquetamab, and elranatamab between 1 August 2022 and 30 September 2024 were retrieved from the Food and Drug Administration’s AE Reporting System (FAERS) database by use of Open Vigil 2.1. AEs were categorized by preferred terms (PTs) and system organ classes (SOCs) as defined by MedDRA. As widely used statistical measures in pharmacovigilance, proportional reporting (PRR) and reporting odds ratios (ROR) were employed to identify potential safety signals.

**Results:**

In total 2,789,182 reports on AEs were retrieved, including 811 for teclistamab, 446 for talquetamab and 302 for elranatamab. Significant associations with immune system disorders, nervous system disorders, benign, malignant and unspecified (incl cysts and polyps) neoplasms, and hepatobiliary disorders were found for all three BsAbs. Common PTs included cytokine release syndrome (CRS), neurotoxicity, immune effector cell-associated neurotoxicity syn-drome (ICANS), pyrexia, and neutropenia. Meanwhile, signal values varied among the three BsAbs. Notably, new safety signals numbered 14, 4, and 5 were identified for teclistamab, talquetamab, and elranatamab, respectively.

**Conclusion:**

Adverse event signals were demonstrated to vary among the three BsAbs used in MM. Significant safety signals identified in the FAERS database which were consistent with previously reported clinical trial data. Furthermore, each BsAb exhibited several novel signals. These findings provide decision-makers and healthcare providers with valuable insights into clinical practice.

## 1 Introduction

Multiple myeloma (MM) is the second most common form of hematologic malignancy, for which a definitive cure remains elusive (1). Recent advancements have introduced several novel therapeutic options for the management of MM, and prominently feature immunotherapies like bispecific antibodies (BsAbs) and chimeric antigen receptor T-cell therapy (2, 3). BsAbs, which engage cytotoxic immune effector cells and tumor cell surface antigens simultaneously, are gaining recognition as a promising category of immunotherapeutic agents in the treatment of MM (4, 5). As of now, three BsAbs have been approved for the treatment of MM (6).

In August 2022, the European Medicines Agency (EMA) granted approval for teclistamab, a BsAb targeting B-cell maturation antigen (BCMA) and cluster of differentiation 3 (CD3) on T cells (7), for treating relapsed or refractory MM (RRMM) patients undergoing at least three prior therapeutic regimens (8). In the phase I/II MajesTEC-1 study, 165 patients whose median was five prior therapy lines received teclistamab, and 39.4% of them achieved a complete response or better. After a median 14.1 months follow-up, the overall response rate (ORR) was reported at 63.0%, and the median length of response was 18.4 months (9).

Akin to teclistamab, elranatamab is a BCMA-CD3 BsAb. It was initially approved in the United States in August 2023 for treating RRMM patients undergoing no less than four prior therapy lines (10). In a phase II clinical trial, elranatamab achieved an ORR of 61.0% (75 out of 123 participants) for the primary endpoint, with a manageable safety profile (11).

Talquetamab as another BsAb targets both CD3 and G-protein coupled receptor family C group five member D (GPRC5D) on T cells (12). It gained accelerated approval in the United States in August 2023 for RRMM patients experiencing failure with four previous therapy lines (13). In the MonumenTAL-1 trial, talquetamab elicited a significant response in RRMM patients receiving at least four prior therapy lines (14).

Despite the transformation of MM treatment by BsAbs, treatment-related adverse events (AEs) are common and distinct (15), with a pooled mortality rate of 0.1% (16). However, these data are primarily derived from clinical trials, which may fail to fully show safety outcomes in real-world clinical practice due to relatively small sample sizes, stringent inclusion criteria, short follow-up periods and other limitations (17). Therefore, it is imperative to investigate the AEs associated with BsAbs in the real world. Among the biggest databases for spontaneous AE reports, the Food and Drug Administration’s AE Reporting System (FAERS) is extensively utilized for evaluating whether drug use is safe in clinical practice (18). In the current study, the FAERS database was used to examine the safety profile of BsAbs approved for MM on a global scale.

## 2 Materials and methods

The data were collected using Open Vigil 2.1 for querying the FAERS database. Open Vigil 2.1, an open-source pharmacovigilance tool designed for the data extraction, cleaning, mining and analysis of the FAERS database, has been utilized in numerous studies (19, 20). Reports on generic names “teclistamab,” “talquetamab,” and “elranatamab” were retrieved between August 2022 and the third quarter of 2024. The clinical features of AE reports involving these study drugs, such as individual safety reports (ISRs), case ID, events, drug name, role code, gender, age, outcomes and reporter country, were gathered. Cases where role code indicated a primary suspect were selected. In this study, ISRs and case IDs were leveraged to eliminate duplicate records in the case of the same case ID, duplicates in the same case were erased, which retained the record with higher ISRs (18). AEs were categorized by preferred terms (PTs) and system organ classes (SOCs) according to version 27.0 of the Medical Dictionary for Regulatory Activities (MedDRA).

### 2.1 Statistical analysis

The clinical features of AE reports related to teclistamab, talquetamab, and elranatamab within the FAERS database were summarized by conducting descriptive statistical analysis. Additionally, disproportionality analysis, including proportional reporting (PRR) and reporting odds ratios (ROR), was performed using Open Vigil 2.1 to identify potential signals (21). The criteria established by Noguchi et al. (22) defined a positive signal of disproportionality as: (1) The 95% confidence interval (CI) of the ROR has a lower limit of greater than 1 and the number of AEs is above or equivalent to 3; (2) The PRR value is above or equivalent to 2 with chi-squared (χ2) value above or equal to 4, and at least three cases. To raise the accuracy of signal analysis and prevent false positives, a signal was classified as positive only if meeting the criteria of both methods. Higher PRR or ROR values indicated that the target drug was strongly statistically associated with AEs. Microsoft Excel 2023 and R (version 4.2.2) were adopted to process data and conduct statistical analyses.

## 3 Results

### 3.1 Descriptive characteristics

From August 2022 to the third quarter of 2024, 2,789,182 AE reports were submitted to FAERS. After deduplication, 1,559 reports associated with BsAbs for MM were included in the analysis and comprised 811 reports for teclistamab, 446 for talquetamab, and 302 for elranatamab. The characteristics of AEs, including gender, age, reporter country and outcomes, are presented in Table 1. Regarding gender distribution, it was observed that talquetamab (121, 27.13%) and elranatamab (132, 43.71%) exhibited a higher proportion of male patients, while teclistamab showed a balanced gender distribution. The majority of cases across all BsAbs were reported in patients at the age of 65 and above. Most cases originated from North America, with 514 (63.38%), 341 (76.46%), and 114 (37.75%) for teclistamab, talquetamab, and elranatamab, respectively. The most commonly reported outcomes were categorized as “other outcomes” for teclistamab (289, 35.64%) and talquetamab (151, 33.86%), whereas the most common outcome for elranatamab (111, 36.75%) was hospitalization.

**TABLE 1:** Characteristics of adverse event (AE) reports associated with teclistamab, talquetamab, and elranatamab.

Characteristic	Teclistamab	Talquetamab	Elranatamab
Number of events	811	446	302
**Gender, *N* (%)**			
Female	263 (32.43)	95 (21.30)	116 (38.41)
Male	258 (31.81)	121 (27.13)	132 (43.71)
Unknown	290 (35.76)	230 (51.57)	54 (17.88)
**Age (years), *N* (%)**			
< 18	0 (0.00)	0 (0.00)	0 (0.00)
18–44	9 (1.11)	7 (1.57)	7 (2.32)
45–64	110 (13.56)	45 (10.09)	82 (27.15)
65–74	117 (14.43)	39 (8.74)	65 (21.52)
≥ 75	121 (14.92)	25 (5.61)	46 (15.23)
Unknown	454 (55.98)	330 (73.99)	102 (33.77)
**Reporter country, *N* (%)**			
North America	514 63.38)	341 (76.46)	114 (37.75)
Asia	14 (1.73)	9 (2.02)	89 (29.47)
Europe	233 (28.73)	74 (16.59)	78 (25.83)
South America	30 (3.70)	13 (2.91)	10 (3.31)
Africa	3 (0.37)	0 (0.00)	0 (0.00)
Oceania	17 (2.10)	9 (2.02)	11 (3.64)
**Outcome, *N* (%)**			
Death	188 (23.18)	28 (6.28)	56 (18.54)
Life-threatening	23 (2.84)	8 (1.79)	8 (2.65)
Disability	9 (1.11)	2 (0.45)	3 (0.99)
Hospitalization (initial or prolonged)	161 (19.85)	67 (15.02)	111 (36.75)
Required intervention	6 (0.74)	0 (0.00)	0 (0.00)
Other outcomes	289 (35.64)	151 (33.86)	79 (26.16)
Unknown	135 (16.65)	190 (42.60)	45 (14.90)

### 3.2 Detection of signals at the SOC level

**FIGURE 1:**
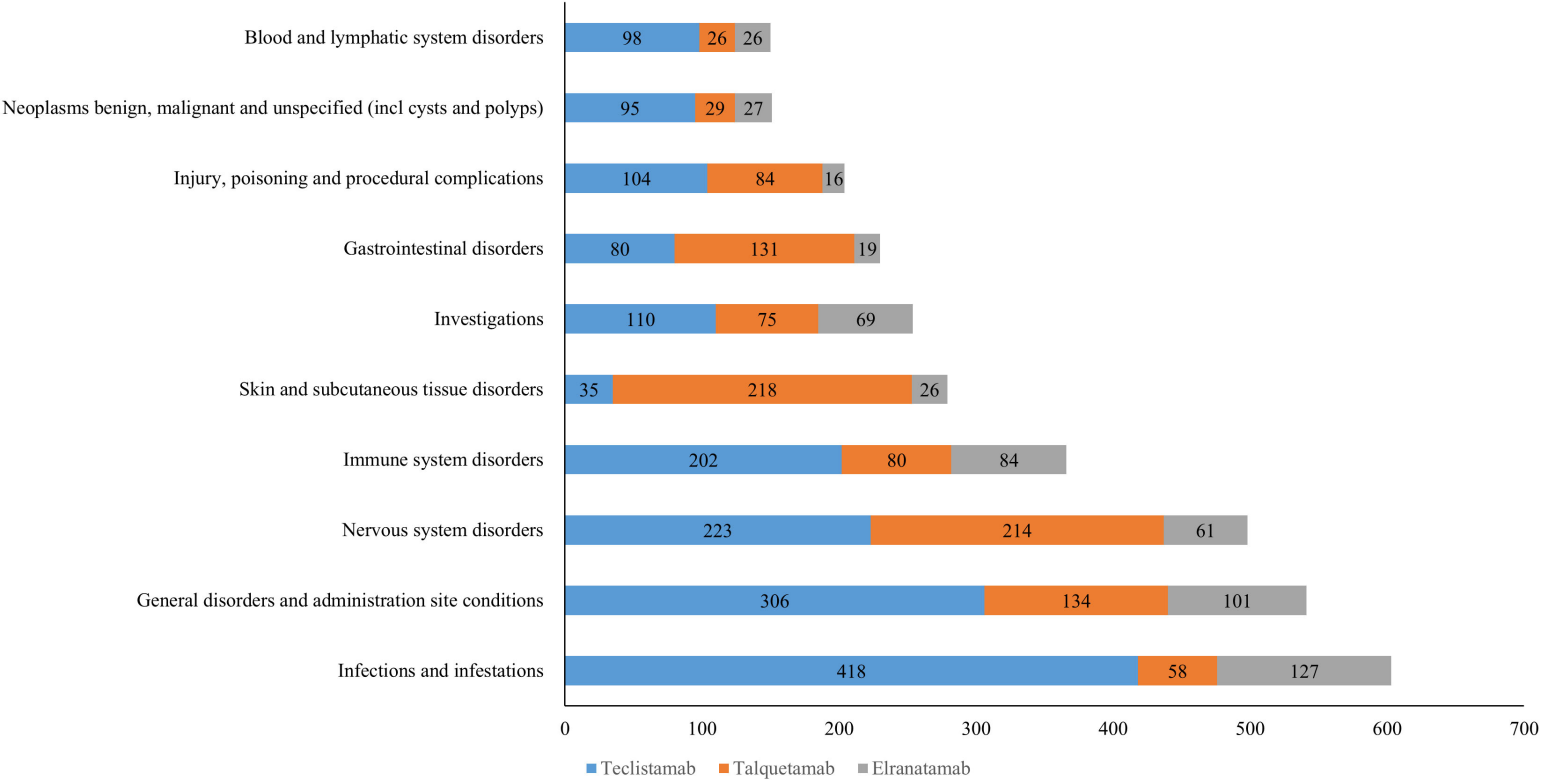
The top 10 most frequently reported system organ classes (SOCs) for teclistamab,talquetamab,and elranatamab.

A total of 25 organ systems were affected by AEs associated with teclistamab, talquetamab, and elranatamab. Among these, four SOCs demonstrated statistically significant associations across all three agents, namely immune system disorders, hepatobiliary disorders, nervous system disorders and neoplasms (benign, malignant and unspecified neoplasms like cysts and polyps). As for signal values, the most pronounced signals for teclistamab were identified in immune system disorders (ROR = 25.78), infections and infestations (ROR = 7.68), and benign, malignant and unspecified neoplasms (including cysts and polyps) (ROR = 7.36). For talquetamab, the strongest signals were observed in immune system disorders (ROR = 20.02), benign, malignant and unspecified neoplasms (including cysts and polyps) (ROR = 15.83), and skin and subcutaneous tissue disorders (ROR = 9.16) after the exclusion of signals related to product issues. Elranatamab exhibited its top three strongest signals in benign, malignant and unspecified neoplasms (including cysts and polyps) (ROR = 32.35), immune system disorders (ROR = 24.95) and eye disorders (ROR = 14.02). The signal strengths for teclistamab, talquetamab, and elranatamab at the SOC level are detailed in Table 2. Furthermore, the 10 most commonly reported SOCs for teclistamab, talquetamab and elranatamab are illustrated in Figure 1. As shown in the figure, infections and infestations, nervous system disorders, immune system disorders, skin and subcutaneous tissue disorders, and general disorders and administration site conditions are the most commonly reported SOCs.

**TABLE 2:** Signal strength for teclistamab, talquetamab, and elranatamab at the system organ class (SOC) level in Food and Drug Administration’s AE Reporting System (FAERS) database.

SOC	Teclistamab		Talquetamab		Elranatamab	
	N	ROR (95% CI)	N	ROR (95% CI)	N	ROR (95% CI)
Vascular disorders	31	1.36 (0.95–1.94)	12	1.21 (0.68–2.15)	6	1.10 (0.49–2.47)
Surgical and medical procedures	28	1.42 (0.97–2.07)	3	0.64 (0.21–2.00)	NA	NA
Social circumstances	1	0.36 (0.05–2.56)	3	1.47 (0.47–4.56)	NA	NA
Skin and subcutaneous tissue disorders	35	0.59 (0.42–0.83)	218	9.16 (7.61–11.03)*	26	1.83 (1.22–2.73)
Respiratory, thoracic and mediastinal disorders	71	1.28 (1.00–1.63)	29	1.11 (0.76–1.62)	23	1.51 (0.99–2.31)
Reproductive system and breast disorders	2	7.67 (1.91–30.78)	1	122.88 (16.94–891.12)	NA	NA
Renal and urinary disorders	25	1.31 (0.88–1.95)	12	1.25 (0.71–2.22)	6	1.62 (0.72–3.64)
Psychiatric disorders	38	0.85 (0.62–1.18)	22	1.23 (0.80–1.89)	5	0.95 (0.39–2.29)
Product issues	3	4.00 (1.29–12.43)*	3	9.28 (2.98–28.92)*	NA	NA
Nervous system disorders	223	2.39 (2.05–2.79)*	214	7.78 (6.46–9.37)*	61	3.07 (2.32–4.07)*
Neoplasms benign, malignant and unspecified (incl cysts and polyps)	95	7.36 (5.94–9.12)*	29	15.83 (10.86–23.08)*	27	32.35 (21.78–48.06)*
Musculoskeletal and connective tissue disorders	49	0.74 (0.56–0.99)	33	0.98 (0.69–1.40)	9	0.86 (0.44–1.66)
Metabolism and nutrition disorders	32	1.47 (1.03–2.10)	34	3.61 (2.55–5.13)*	21	3.04 (1.95–4.73)*
Investigations	110	1.78 (1.45–2.17)	75	3.20 (2.49–4.10)*	69	4.55 (3.48–5.95)*
Injury, poisoning and procedural complications	104	0.61 (0.49–0.74)	84	1.00 (0.79–1.26)	16	0.59 (0.35–0.97)
Infections and infestations	418	7.68 (6.69–8.81)*	58	1.58 (1.20–2.08)	127	6.29 (5.01–7.91)*
Immune system disorders	202	25.78 (21.98–30.23)*	80	20.02 (15.72–25.51) *	84	24.95 (19.40–32.10)*
Hepatobiliary disorders	12	2.46 (1.39–4.34) *	5	2.79 (1.15–6.73)*	6	4.47 (1.99–10.04)*
General disorders and administration site conditions	306	0.96 (0.83–1.10)	134	0.79 (0.64–0.97)	101	1.50 (1.18–1.90)
Gastrointestinal disorders	80	0.68 (0.54–0.86)	131	2.54 (2.07–3.11) *	19	0.58 (0.36–0.92)
Eye disorders	15	0.82 (0.49–1.36)	5	5.16 (2.14–12.47)*	11	14.02 (7.67–25.60)*
Ear and labyrinth disorders	3	1.13 (0.36–3.50)	NA	NA	1	36.62 (5.12–261.83)
Cardiac disorders	35	1.35 (0.96–1.89)	7	1.41 (0.67–2.98)	8	1.79 (0.89–3.62)
Blood and lymphatic system disorders	98	3.87 (3.13–4.78)*	26	1.88 (1.26–2.79)	26	2.78 (1.86–4.15)*
Endocrine disorders	NA	NA	1	2.65 (0.37–18.85)	NA	NA

*Indicates significant signals in the algorithm. NA, not available.

### 3.3 Detection of signals at the PT level

**FIGURE 2:**
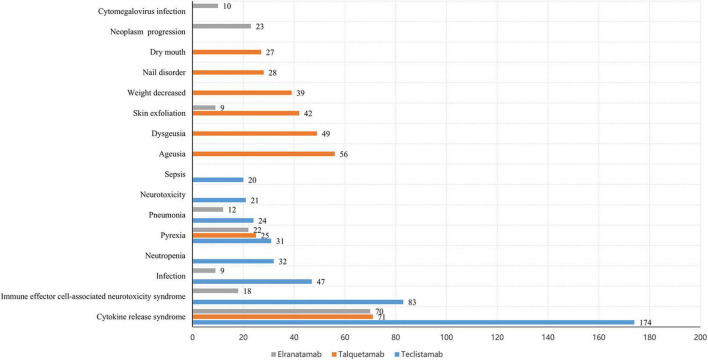
The top 8 most frequently reported adverse events (AEs) associated with teclistamab, talquetamab, and elranatamab at the preferred term (PT) level.

**FIGURE 3:**
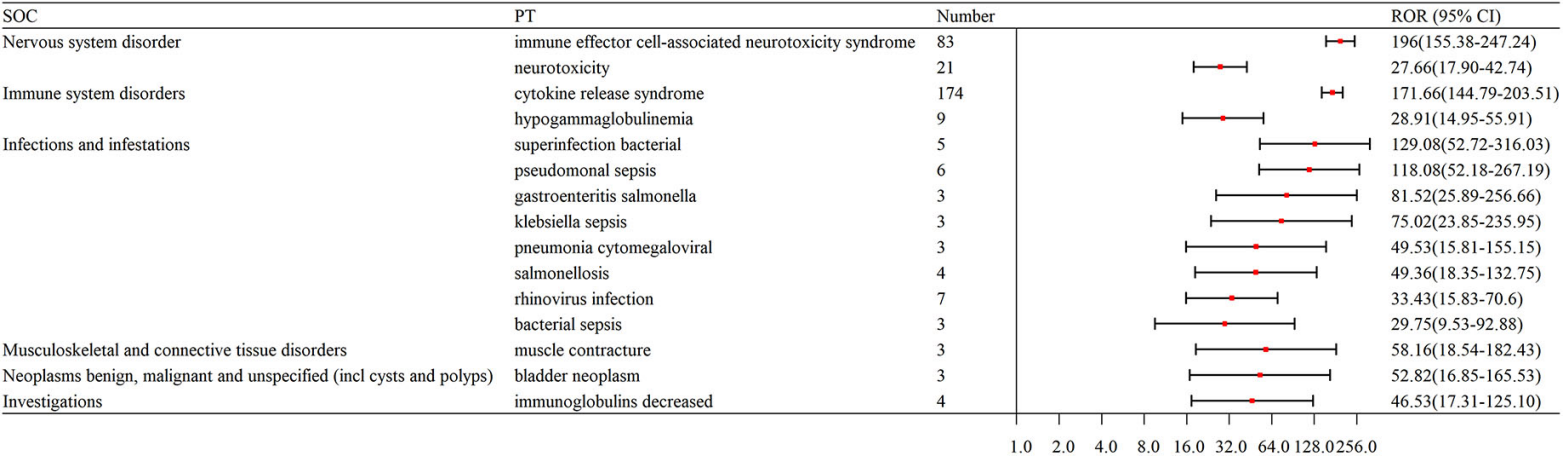
The top 15 strongest signals for teclistamab at the preferred term (PT) level.

**FIGURE 4:**
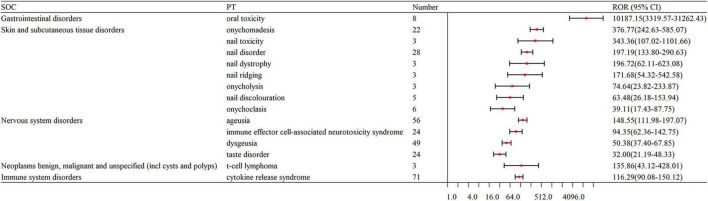
The top 15 strongest signals for talquetamab at the preferred term (PT) level.

**FIGURE 5:**
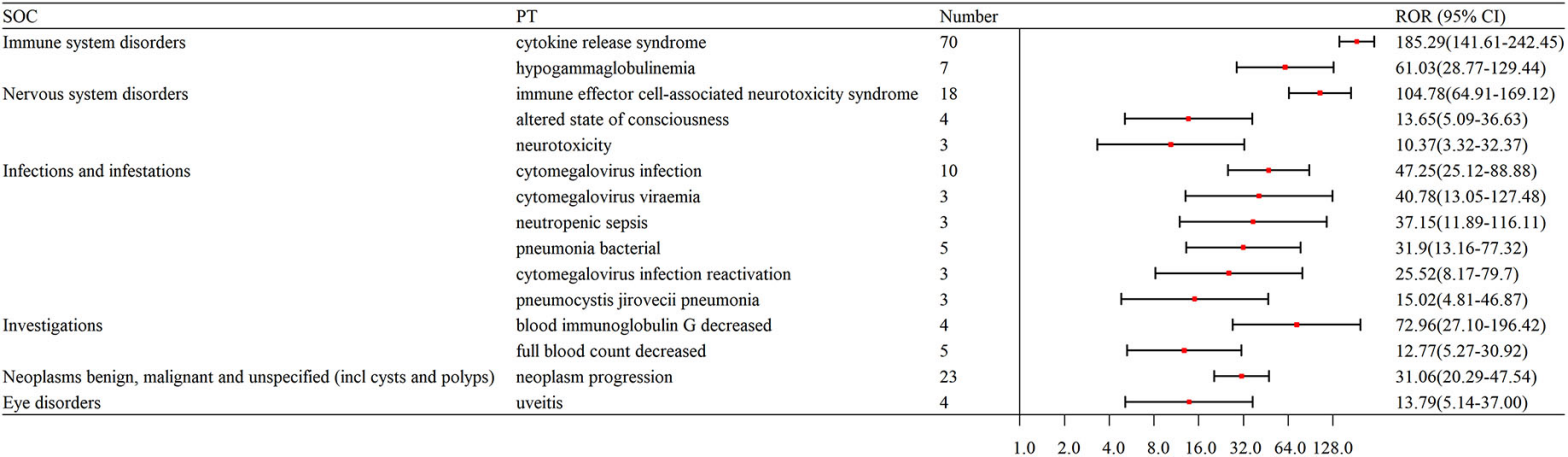
The top 15 strongest signals for elranatamab at the preferred term (PT) level.

The number of remarkable PT signals identified for teclistamab, talquetamab, and elranatamab was 73, 47, and 32, respectively. After signals related to AEs, adverse reactions, adverse drug reactions, product selection errors and other factors of limited research significance were excluded, 69 PTs for teclistamab, 42 PTs for talquetamab, and 32 PTs for elranatamab were retained as drug-related and statistically significant. The top 8 most commonly reported AEs associated with teclistamab, talquetamab, and elranatamab at the PT level are demonstrated in Figure 2. The top 15 strongest signals meeting the established criteria are presented in Figures 3–5.

### 3.4 ROR for the same PTs with teclistamab, talquetamab, and elranatamab

The signal strengths for teclistamab, talquetamab, and elranatamab for the same PTs are presented in Table 3. Five PTs were statistically significant across all these three BsAbs: neurotoxicity, pyrexia, neutropenia, cytokine release syndrome (CRS) and immune effector cell-associated neurotoxicity syndrome (ICANS). In the meantime, signal values varied among the three BsAbs. For teclistamab, the stronger signals were observed in neutropenia (*N* = 32, ROR = 5.04), ICANS (*N* = 83, ROR = 196.00), infections (*N* = 47, ROR = 8.38), neurotoxicity (*N* = 21, ROR = 27.66) and bacteremia (*N* = 6, ROR = 13.08). Talquetamab demonstrated stronger signals in skin exfoliation (*N* = 42, ROR = 26.29), bone pain (*N* = 6, ROR = 5.83), nervous system disorders (*N* = 3, ROR = 8.40) and decreased immune responsiveness (*N* = 4, ROR = 13.91). Elranatamab exhibited notable signals in pancytopenia (*N* = 5, ROR = 6.70), pyrexia (*N* = 22, ROR = 5.55), chills (*N* = 5, ROR = 3.34), CRS (*N* = 70, ROR = 185.29), hypogammaglobulinemia (*N* = 7, ROR = 61.03), c-reactive protein increased (*N* = 5, ROR = 10.29), and pneumonia (*N* = 12, ROR = 2.90), cytomegalovirus infection reactivation (*N* = 3, ROR = 25.52), viral infection (*N* = 3, ROR = 6.28), pneumocystis jirovecii pneumonia (*N* = 3, ROR = 15.02), pneumonia bacterial (*N* = 5, ROR = 31.90) and cytomegalovirus infection (*N* = 10, ROR = 47.25).

**TABLE 3:** Signal strength for teclistamab, talquetamab, and elranatamab from Food and Drug Administration’s AE Reporting System (FAERS) in the same preferred terms (PTs).

PT	Teclistamab		Talquetamab		Elranatamab	
	N	ROR (95% CI)	N	ROR (95% CI)	N	ROR (95% CI)
Immune effector cell-associated neurotoxicity syndrome	83	196 (155.38–247.24)*	24	94.35 (62.36–142.75)*	18	104.78 (64.91–169.12)*
Neurotoxicity	21	27.66 (17.90–42.74)*	4	9.36 (3.49–25.07)*	3	10.37 (3.32–32.37)*
Nervous system disorder	3	4.61 (1.48–14.32)*	3	8.40 (2.70–26.17)*	1	4.12 (0.58–29.35)
Cytokine release syndrome	174	171.66 (144.79–203.51)*	71	116.29 (90.08–150.12)*	70	185.29 (141.61–242.45)*
Hypogammaglobulinemia	9	28.91 (14.95–55.91)*	2	11.53 (2.87–46.31)	7	61.03 (28.77–129.44)*
Decreased immune responsiveness	3	5.70 (1.83–17.74)*	4	13.91 (5.19–37.27)*	NA	NA
Pyrexia	31	2.81 (1.96–4.02)*	25	4.19 (2.80–6.28)*	22	5.55 (3.60–8.57)*
Chills	10	2.48 (1.33–4.62)*	4	1.80 (0.67–4.81)	5	3.34 (1.38–8.09)*
Neutropenia	32	5.04 (3.54–7.18)*	8	2.24 (1.11–4.51)*	7	2.91 (1.37–6.16)*
Pancytopenia	6	2.96 (1.33–6.62)*	2	1.79 (0.45–7.18)	5	6.70 (2.77–16.21)*
Infection	47	8.38 (6.24–11.26)*	6	1.85 (0.83–4.15)	9	4.18 (2.15–8.11)*
Pneumonia	24	2.14 (1.42–3.21)*	3	0.47 (0.15–1.48)	12	2.90 (1.63–5.17)*
Cytomegalovirus infection reactivation	8	25.45 (12.65–51.2)*	1	5.71 (0.8–40.63)	3	25.52 (8.17–79.70)*
Bacteremia	6	13.08 (5.85–29.25)*	3	11.86 (3.81–36.96)*	2	11.67 (2.90–46.93)
Viral infection	6	4.67 (2.09–10.43) *	NA	NA	3	6.28 (2.01–19.59) *
Pneumocystis jirovecii pneumonia	5	9.29 (3.85–22.42)*	1	3.36 (0.47–23.92)	3	15.02 (4.81–46.87)*
Pneumonia bacterial	4	9.38 (3.51–25.10)*	NA	NA	5	31.90 (13.16–77.32)*
Cytomegalovirus infection	3	5.10 (1.64–15.87)*	1	3.09 (0.43–21.97)	10	47.25 (25.12–88.88)*
Skin exfoliation	NA	NA	42	26.29 (19.12–36.14)*	9	7.74 (3.99–15.04)*
Bone pain	7	3.72 (1.77–7.83)*	6	5.83 (2.60–13.05)*	1	1.42 (0.20–10.11)
C–reactive protein increased	8	6.09 (3.04–12.24)*	2	2.75 (0.69–11.04)	5	10.29 (4.25–24.92)*

*Indicates significant signals in the algorithm. NA, not available.

### 3.5 New signals

After an analysis of drug labels, we identified 14, 4, and 5 novel signals for teclistamab, talquetamab, and elranatamab, respectively. A detailed comparison of novel versus known signals at the same SOC across the three BsAbs is presented in Table 4. For teclistamab, the five most prominent signals encompassed bacterial superinfection (*N* = 5, ROR = 129.08), gastroenteritis salmonella (*N* = 3, ROR = 81.52), bladder neoplasm (*N* = 3, ROR = 52.82), necrotizing fasciitis (*N* = 3, ROR = 22.75) and bronchopulmonary aspergillosis (*N* = 5, ROR = 15.04). The four strongest signals for talquetamab were t-cell lymphoma (*N* = 3, ROR = 135.86), mucosal inflammation (*N* = 10, ROR = 20.78), anosmia (*N* = 4, ROR = 19.86) and decreased immune responsiveness (*N* = 4, ROR = 13.91). The five strongest signals for elranatamab included increased c-reactive protein (*N* = 5, ROR = 10.29), uveitis (*N* = 4, ROR = 13.79), decreased red blood cell count (*N* = 4, ROR = 9.02), cellulitis (*N* = 3, ROR = 5.06) and respiratory failure (*N* = 3, ROR = 4.10).

**TABLE 4:** Comparison of novel vs. known signals at the same system organ class (SOC) across the three bispecific antibodies (BsAbs).

Drug name	SOC	PT	N	ROR (95% CI)	PRR (χ2)
Teclistamab	Infections and infestations	Infection	47	8.38 (6.24–11.26)	7.95 (280.22)
		Pneumonia	24	2.14 (1.42–3.21)	2.1 (12.99)
		Sepsis	20	5.39 (3.46–8.4)	5.28 (65.36)
		Septic shock	11	7.06 (3.89–12.8)	6.97 (50.47)
		Upper respiratory tract infection	9	5.41 (2.8–10.45)	5.36 (27.74)
		Cytomegalovirus infection reactivation	8	25.45 (12.65–51.2)	25.21 (161.46)
		Rhinovirus infection	7	33.43 (15.83–70.6)	33.15 (185.55)
		Pseudomonal sepsis	6	118.08 (52.18–267.19)	117.21 (560.88)
		Bacteremia	6	13.08 (5.85–29.25)	12.99 (54.78)
		COVID-19 pneumonia	6	6.3 (2.82–14.08)	6.26 (21.51)
		Viral infection	6	4.67 (2.09–10.43)	4.64 (13.69)
		Superinfection bacterial*	5	129.08 (52.72–316.03)	128.29 (492.24)
		Bronchopulmonary aspergillosis*	5	15.04 (6.23–36.29)	14.95 (51.67)
		Pneumocystis jirovecii pneumonia	5	9.29 (3.85–22.42)	9.24 (28.91)
		Clostridium difficile infection*	5	5.14 (2.13–12.38)	5.11 (12.67)
		Respiratory tract infection	5	5.06 (2.1–12.2)	5.04 (12.38)
		Salmonellosis	4	49.36 (18.35–132.75)	49.12 (141.48)
		Pyelonephritis	4	13.82 (5.16–36.97)	13.75 (35.28)
		Progressive multifocal leukoencephalopathy*	4	13.28 (4.96–35.55)	13.22 (33.68)
		Urosepsis	4	12.99 (4.85–34.75)	12.93 (32.78)
		Pseudomonas infection	4	12.72 (4.75–34.04)	12.66 (31.99)
		Pneumonia bacterial	4	9.38 (3.51–25.1)	9.34 (21.99)
		Gastroenteritis salmonella*	3	81.52 (25.89–256.66)	81.22 (160.44)
		Klebsiella sepsis	3	75.02 (23.85–235.95)	74.74 (147.54)
		Pneumonia cytomegaloviral	3	49.53 (15.81–155.15)	49.35 (96.49)
		Bacterial sepsis	3	29.75 (9.53–92.88)	29.64 (56.37)
		Necrotizing fasciitis*	3	22.75 (7.3–70.95)	22.67 (42.08)
		Post-acute COVID-19 syndrome	3	18.99 (6.09–59.2)	18.93 (34.4)
		Herpes virus infection	3	15.75 (5.06–49.08)	15.7 (27.78)
		Escherichia infection	3	10.05 (3.23–31.27)	10.01 (16.12)
		Aspergillus infection	3	9.76 (3.14–30.38)	9.73 (15.54)
		Cytomegalovirus infection	3	5.1 (1.64–15.87)	5.09 (6.19)
		Bacterial infection	3	4.2 (1.35–13.04)	4.18 (4.43)
	Gastrointestinal disorders	Colitis*	10	6.36 (3.41–11.87)	6.30 (39.4)
	Immune system disorders	Cytokine release syndrome	174	171.66 (144.79–203.51)	135.04 (22183.78)
		Hypogammaglobulinemia	9	28.91 (14.95–55.91)	28.6 (211.22)
		Hemophagocytic lymphohistiocytosis*	6	10.73 (4.8–23.99)	10.66 (43.21)
		Immunosuppression*	3	7.05 (2.27–21.94)	7.03 (10.06)
		Decreased immune responsiveness*	3	5.7 (1.83–17.74)	5.69 (7.37)
	Investigations	Neutrophil count decreased	9	4.75 (2.46–9.18)	4.71 (22.76)
		C-reactive protein increased*	8	6.09 (3.04–12.24)	6.04 (28.82)
		Immunoglobulins decreased	4	46.53 (17.31–125.1)	46.31 (133.11)
		Blood lactic acid increased*	3	13.17 (4.23–41)	13.12 (22.49)
		General physical condition abnormal	3	11.05 (3.55–34.38)	11.01 (18.15)
	Neoplasms benign, malignant and unspecified (incl cysts and polyps)	Bladder neoplasm*	3	52.82 (16.85–165.53)	52.63 (103.11)
	Respiratory, thoracic and mediastinal disorders	Pulmonary embolism*	6	2.71 (1.21–6.05)	2.7 (4.83)
		acute respiratory failure	3	4.21 (1.35–13.09)	4.2 (4.46)
		Respiratory distress	3	4.2 (1.35–13.05)	4.19 (4.44)
Talquetamab	Neoplasms benign, malignant and unspecified (incl cysts and polyps)	T-cell lymphoma*	3	135.86 (43.12–428.01)	134.95 (270.33)
	General disorders and administration site conditions	Pyrexia	25	4.19 (2.8–6.28)	4.02 (54.36)
		Mucosal inflammation*	10	20.78 (11.09–38.94)	20.34 (164.71)
	Nervous system disorders	Ageusia	56	148.55 (111.98–197.07)	130.02 (6904.69)
		Dysgeusia	49	50.38 (37.4–67.85)	44.95 (2052.29)
		Taste disorder	24	32.00 (21.19–48.33)	30.34 (649.82)
		Immune effector cell-associated neurotoxicity syndrome	24	94.35 (62.36–142.75)	89.33 (1981.59)
		Neurotoxicity	4	9.36 (3.49–25.07)	9.28 (21.85)
		Anosmia*	4	19.86 (7.41–53.24)	19.69 (53.37)
	Immune system disorders	Cytokine release syndrome	71	116.29 (90.08–150.12)	97.94 (6622.99)
		Decreased immune responsiveness*	4	13.91 (5.19–37.27)	13.80 (35.48)
Elranatamab	Respiratory, thoracic and mediastinal disorders	Respiratory failure*	3	4.10 (1.31–12.78)	4.07 (4.22)
	Investigations	Platelet count decreased	6	3.78 (1.68–8.48)	3.72 (9.44)
		Full blood count decreased	5	12.77 (5.27–30.92)	12.57 (42.31)
		C-reactive protein increased*	5	10.29 (4.25–24.92)	10.14 (32.57)
		Blood immunoglobulin G decreased	4	72.96 (27.1–196.42)	72.01 (211.95)
		Red blood cell counts decreased*	4	9.02 (3.36–24.19)	8.91 (20.74)
		Aspartate aminotransferase increased	3	6.12 (1.96–19.08)	6.07 (8.14)
		Alanine aminotransferase increased	3	5.14 (1.65–16.03)	5.1 (6.22)
	Eye disorders	Uveitis*	4	13.79 (5.14–37.00)	13.62 (34.97)
	Infections and infestations	Pneumonia	12	2.9 (1.63–5.17)	2.82 (12.55)
		Cytomegalovirus infection	10	47.25 (25.12–88.88)	45.72 (392.15)
		Infection	9	4.18 (2.15–8.11)	4.08 (18.11)
		Pneumonia bacterial	5	31.9 (13.16–77.32)	31.39 (117.94)
		Cytomegalovirus viremia	3	40.78 (13.05–127.48)	40.39 (78.88)
		Neutropenic sepsis	3	37.15 (11.89–116.11)	36.79 (71.46)
		Cytomegalovirus infection reactivation	3	25.52 (8.17–79.7)	25.28 (47.67)
		Pneumocystis jirovecii pneumonia	3	15.02 (4.81–46.87)	14.88 (26.17)
		Viral infection	3	6.28 (2.01–19.59)	6.23 (8.46)
		Cellulitis*	3	5.06 (1.62–15.78)	5.02 (6.06)

*Indicates preferred terms (PTs) that are not listed on the drug label.

## 4 Discussion

In this pharmacovigilance study, data from the FAERS database were employed to investigate the actual safety profiles of BsAbs in MM. The results indicated that teclistamab, talquetamab and elranatamab were consistently associated with CRS, neurotoxicity, ICANS and neutropenia. Meanwhile, infectious complications were prevalent with BsAbs in MM. The study also revealed that AE signals varied among the three BsAbs. Furthermore, teclistamab, and elranatamab were associated with an increased risk of infections and neutropenia compared to talquetamab, while nail disorders and skin changes had been specifically linked to talquetamab. These findings were consistent with prior clinical trial data.

### 4.1 CRS

Cytokine release syndrome is a commonly reported adverse reaction associated with BsAbs. Characterized as a systemic inflammatory response, CRS typically arises from the on-target effects of BsAbs binding simultaneously to their antigenic targets on effector and plasma cells, which leads to the release of cytokines like Interferon-gamma (IFN-γ) and tumor necrosis factor-alpha (TNF-α) (23). These cytokines activate both immune and non-immune cells, which results in a significant release of additional cytokines (24). The findings indicated that CRS was the most frequently reported adverse reaction for BsAbs in MM at the PT level. Specifically, CRS was reported in 174 cases with teclistamab, 70 cases with elranatamab, and 71 cases with talquetamab. A recent meta-analysis suggested that the incidence of CRS in RRMM patients treated with BsAbs was 59% (95% CI: 49%–68%) (25). In the phase 1/2 MajesTEC-1 study, it was observed that a majority of CRS cases occurred during the step-up dosing schedule for BsAbs and were classified as grade 1 or 2 (26). It suggested that the incidence of CRS of grade ≥ 3 was lower for BsAb therapy administered subcutaneously (SC) compared to intravenously (IV) (25). The use of tocilizumab or other interleukin-6 (IL-6)/IL-6 receptor inhibitors is recommended for the management of CRS following cooperative group guidelines (27).

### 4.2 Neurotoxicity including ICANS

Neurotoxicity represents a class effect associated with BsAbs and often manifests as ICANS within the initial days after the infusion or administration of initial doses (28). ICANS features symptoms such as confusion, focal neurological deficits and seizures (29). In this study, obvious neurotoxic signals, including ICANS, have been observed with these three BsAbs in MM. ICANS emerged as one of the top 15 strongest signals for teclistamab (*N* = 83, ROR = 196), elranatamab (*N* = 18, ROR = 104.78), and talquetamab (*N* = 24, ROR = 94.35). In alignment with the findings of this study, the MajesTEC-1 trial showed overall neurotoxicity in 14.5% of patients treated with teclistamab, with ICANS occurring in five patients (3%). Neurotoxic events were mostly categorized as grade 1–2, with ICANS being entirely grade 1–2 (9). Similarly, approximately 3.4% of patients experienced ICANS after elranatamab therapy (30), and ICANS was detected in around 10% of patients who received talquetamab at a dose of 405 μg (14). Notwithstanding this association, the precise pathogenesis of BsAbs-induced neurotoxicity remains unclear and is considered as an off-target effect. One proposed mechanism is that circulating cytokines may exert a direct influence on the central nervous system by activating endothelial cells and compromising the integrity of the blood-brain barrier (31, 32). The primary treatments for ICANS include glucocorticoids, with anakinra and tocilizumab as alternative options when CRS is also present (15).

### 4.3 Hemotoxicity with cytopenias

Hemotoxicity, another prevalent AE associated with BsAbs in MM, is hallmarked by cytopenias, which represent the most frequent grade ≥ 3 toxicities (6). A meta-analysis demonstrated that patients experienced a higher frequency of hematologic AEs in comparison with non-hematologic events, with anemia, neutropenia and thrombocytopenia being the most prevalent (17). Likewise, another meta-analysis documented a higher incidence of hematologic toxicity in patients treated with BsAbs for MM, including neutropenia (12%–75%), anemia (5%–52%), and thrombocytopenia (14%–42%) (16). One study further indicated that BCMA-targeted BsAb therapy posed a greater risk of neutropenia compared with non-BCMA-targeted BsAb therapy (33). In line with these findings, our research identified a stronger association between neutropenia and treatment with teclistamab and elranatamab compared to talquetamab. It is thought that the mechanism underlying BsAbs-associated hematologic toxicity involves cytokines released by the bone marrow microenvironment and suppressing hematopoiesis (34). Thus, the management of BsAbs-related cytopenias can be facilitated via supportive interventions like transfusion, and the use of bone marrow-stimulating agents like erythropoiesis-stimulating agents and granulocyte colony-stimulating factors (G-CSFs) (29).

### 4.4 Hypogammaglobulinemia and infections

In this study, it was identified that BCMA-targeting BsAbs were significantly related to hypogammaglobulinemia. Specifically, teclistamab (*N* = 9, ROR = 28.91) and elranatamab (*N* = 7, ROR = 61.03) exhibited a stronger correlation with this adverse event, whereas talquetamab (*N* = 2, ROR = 11.53) showed a comparatively lower association. Consistent with the findings of this study, a phase 1–2 study reported that 123 (74.5%) patients developed hypogammaglobulinemia when treated with teclistamab (9). Another study also confirmed that hypogammaglobulinemia was a risk factor for infections associated with BsAbs (35). Furthermore, factors such as T-cell dysfunction, low bone marrow reserves and prolonged cytopenias, particularly neutropenia, which result from primary disease and previous therapies, may exacerbate the risk of infections (36).

Based on this study, infections and infestations were the most common BsAbs-related AEs in MM at the SOC level. Clinical trials that involved teclistamab, talquetamab, and elranatamab reported grade 3/4 infection rates ranging from 7% to 55.2% (14, 30, 37). A meta-analysis of 1,666 patients with BsAbs in MM from 16 clinical trials revealed a grade ≥ 3 infection rate of 24% (38). Consequently, it is pressing to implement preventive strategies for patients treated with BsAbs for MM to mitigate the risk of infections (39, 40). It is advisable for patients, especially neutropenic patients experiencing grade 3 infections, to receive vaccinations, drug prophylaxis, intravenous immunoglobulin and colony-stimulating factors (41).

Our study demonstrated that teclistamab and elranatamab were more strongly associated with infections at the PT level compared to talquetamab. Consistent with our findings,a pharmacovigilance study revealed that anti-BCMA BsAbs were linked to a 2-fold rise in the risk of infectious complications relative to other MM treatments (42). Similarly, a systematic review identified that BCMA-targeted bispecifics had a higher risk of infection than non-BCMA targeted bispecifics (38).

### 4.5 Skin- and nail-related AEs

In this study, the skin and nail related AEs of talquetamab were significantly stronger than those of teclistamab and elranatamab, including nail disorders (*N* = 28, ROR = 197.19) and skin exfoliation (*N* = 42, ROR = 26.29), which indicated an increased risk of skin- and nail-related AEs for talquetamab. This difference in toxicity profiles can be attributed to the distinct molecular targets of BsAbs. Talquetamab targeting GPRC5D is believed to affect keratin-containing tissues in that GPRC5D is expressed in these tissues (43, 44). By comparison, the association between skin-related AEs and BCMA-targeted BsAbs is less well understood. Consistent with this research, a phase I clinical trial showed that 65% of patients administered talquetamab experienced skin-related AEs (45). Furthermore, a recent retrospective study of 14 patients reported hand-foot syndrome in 50% of the participants (46). Management strategies for these AEs include the topical application of moisturizing lotions and topical corticosteroids (15, 47).

### 4.6 Others

In this study, the most commonly reported AEs for teclistamab, talquetamab, and elranatamab include CRS, neurotoxicity, infections and neutropenia, all of which are listed as common adverse drug reactions (ADRs) in the prescribing information of drugs. Nevertheless, it is noteworthy that musculoskeletal pain is also classified as a common ADR in prescribing information, with a reported occurrence frequency of ≥ 20%. In contrast, the findings of this study indicate a relatively low frequency of musculoskeletal pain. To be specific, teclistamab was correlated with seven cases of bone pain (ROR = 3.72), and talquetamab was associated with nine cases of back pain (ROR = 2.14) and six cases of bone pain (ROR = 5.82), whereas no significant signal was identified for elranatamab. Consistent with the research findings, no AEs related to musculoskeletal pain were reported in a real-world analysis of teclistamab in 123 relapsed/refractory MM patients from Germany (48). This discrepancy may be ascribed to the fact that patients may attribute musculoskeletal pain to their underlying disease and may not actively report it in real-world clinical settings. Furthermore, healthcare providers may prescribe alternative medications to ease musculoskeletal pain, which could have an impact on the frequency of AE reporting.

New signals were noted for each BsAb used for MM, including T-cell lymphoma with talquetamab (*N* = 3, ROR = 135.86) and bladder neoplasm with teclistamab (*N* = 3, ROR = 52.82). This study reveals that BsAb therapy for MM is linked to a high risk of secondary primary malignancies (SPMs), but in a small sample. Similarly to this study, Liang et al. discovered that BsAb therapy was related to a high risk of SPMs (49). Braun T reported a case of a patient who experienced the early relapse of his MM after anti-BCMA CART cell therapy and developed a special T cell neoplasm after salvage treatment with talquetamab (50). The mechanisms underlying SPMs after BsAb therapy may result from prior treatments weakening the immune system (51). In addition, BsAbs recruit immune cells capable of depleting the T cells needed to monitor and control SPMs. BsAbs-induced immune overactivation can lead to the exhaustion of immune cells, which further increases the risk of SPMs (52). Chronic inflammation caused by BsAb therapy is also likely to create a tumor-friendly microenvironment promoting the growth of malignant clones (53). However, it is necessary to confirm these hypotheses through future research. Beyond that, it is recommended that patients receiving these therapies and those in clinical trials undergo regular monitoring for the emergence of SPMs.

### 4.7 Limitations

This analysis is subject to several limitations that warrant discussion. Firstly, the FAERS database is dependent on self-reported data and contains a number of missing records, which may lead to potentially misleading results (54). Secondly, the FAERS database is limited to patients with AEs and does not provide an estimate of the total number of patients receiving BsAbs in MM, which thus complicates the estimation of AE incidence rates (20). Nevertheless, relative safety can be inferred by making a comparison between the signal strength of drugs and similar therapeutic indications or pharmacological mechanisms. Thirdly, disprotionality analysis only reveals statistical associations, which makes it impossible to establish causality between the target drug and AE (54). As a result, it is important to take these limitations into account when interpreting the findings of this research. Further studies are required to validate the findings.

## 5 Conclusion

In this study, the FAERS database was used to examine the safety profiles of BsAbs used in MM. The findings indicate the associations of teclistamab, talquetamab, and elranatamab with elevated rates of CRS, neurotoxicity, ICANS and neutropenia. Meanwhile, infectious complications are prevalent with BsAbs in MM. Furthermore, nail disorders and skin changes are specific to talquetamab. Additionally, novel safety signals were identified for each BsAb, which may influence drug monitoring and clinical practice. Overall, this study offers valuable insights into the AEs of BsAbs in the real world, which aligns with findings from clinical trials. Future research should aim to clarify the pathophysiology of these toxicities and create evidence-based strategies for their management.

## Data Availability

The datasets presented in this study can be found in online repositories. The names of the repository/repositories and accession number(s) can be found in the article/supplementary material.
